# 2-[5-Methyl-2-(propan-2-yl)phen­oxy]-*N*′-{2-[5-methyl-2-(propan-2-yl)phen­oxy]acet­yl}acetohydrazide

**DOI:** 10.1107/S1600536811033964

**Published:** 2011-08-27

**Authors:** Hoong-Kun Fun, Ching Kheng Quah, Nithinchandra, Balakrishna Kalluraya

**Affiliations:** a X-ray Crystallography Unit, School of Physics, Universiti Sains Malaysia, 11800 USM, Penang, Malaysia; b Department of Studies in Chemistry, Mangalore University, Mangalagangotri, Mangalore 574 199, India

## Abstract

The complete mol­ecule of the title compound, C_24_H_32_N_2_O_4_, is generated by a crystallographic inversion center. The 1,2-diethyl­hydrazine moiety is nearly planar, with a maximum deviation of 0.024 (1) Å, and is inclined at a dihedral angle of 54.20 (4)° with the phenyl ring. In the crystal, [001] chains are formed, with adjacent mol­ecules in the chain linked by pair of inter­molecular N—H⋯O hydrogen bonds, generating *R* ^2^_2_(10) ring motifs. Inter­molecular C—H⋯O hydrogen bonds and C—H⋯π inter­actions are also observed.

## Related literature

For general background to and the biological activity of hydrazides, see: Bedia *et al.* (2006 ▶); Rollas *et al.* (2002 ▶); Terzioglu & Gürsoy (2003 ▶); Bratenko *et al.* (1999 ▶); Rai *et al.* (2008 ▶). For standard bond-length data, see: Allen *et al.* (1987 ▶). For hydrogen-bond motifs, see: Bernstein *et al.* (1995 ▶). For the stability of the temperature controller used for the data collection, see: Cosier & Glazer (1986 ▶).
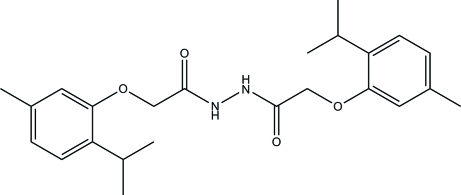


## Experimental

### 

#### Crystal data

C_24_H_32_N_2_O_4_
*M* *_r_* = 412.52
Orthorhombic, 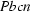

*a* = 23.6018 (8) Å
*b* = 11.2077 (4) Å
*c* = 8.6653 (3) Å
*V* = 2292.16 (14) Å^3^
*Z* = 4
Mo *K*α radiation
μ = 0.08 mm^−1^
*T* = 100 K
0.98 × 0.23 × 0.18 mm

#### Data collection

Bruker SMART APEXII CCD diffractometer
Absorption correction: multi-scan (*SADABS*; Bruker, 2009 ▶) *T*_min_ = 0.914, *T*_max_ = 0.986
38738 measured reflections
3337 independent reflections
2946 reflections with *I* > 2σ(*I*)
*R*_int_ = 0.030

#### Refinement

*R*[*F* ^2^ > 2σ(*F* ^2^)] = 0.041
*wR*(*F* ^2^) = 0.111
*S* = 1.04
3337 reflections
143 parameters
H atoms treated by a mixture of independent and constrained refinement
Δρ_max_ = 0.34 e Å^−3^
Δρ_min_ = −0.21 e Å^−3^

### 

Data collection: *APEX2* (Bruker, 2009 ▶); cell refinement: *SAINT* (Bruker, 2009 ▶); data reduction: *SAINT*; program(s) used to solve structure: *SHELXTL* (Sheldrick, 2008 ▶); program(s) used to refine structure: *SHELXTL*; molecular graphics: *SHELXTL*; software used to prepare material for publication: *SHELXTL* and *PLATON* (Spek, 2009 ▶).

## Supplementary Material

Crystal structure: contains datablock(s) global, I. DOI: 10.1107/S1600536811033964/hb6377sup1.cif

Structure factors: contains datablock(s) I. DOI: 10.1107/S1600536811033964/hb6377Isup2.hkl

Supplementary material file. DOI: 10.1107/S1600536811033964/hb6377Isup3.cml

Additional supplementary materials:  crystallographic information; 3D view; checkCIF report

## Figures and Tables

**Table 1:** Hydrogen-bond geometry (Å, °) *Cg*1 is the centroid of the C1–C6 phenyl ring.

*D*—H⋯*A*	*D*—H	H⋯*A*	*D*⋯*A*	*D*—H⋯*A*
N1—H1*N*1⋯O2^i^	0.902 (16)	1.916 (15)	2.7759 (11)	158.8 (13)
C11—H11*A*⋯O2^ii^	0.96	2.58	3.4830 (14)	157
C7—H7*B*⋯*Cg*1^iii^	0.97	2.68	3.3706 (10)	129

Symmetry codes: (i) 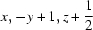; (ii) 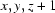; (iii) 
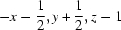
.

**Fig. 1.**
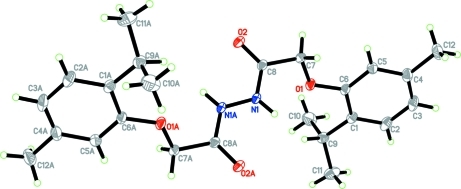
The molecular structure of the title compound showing 50% probability displacement ellipsoids for non-H atoms.

**Fig. 2.**
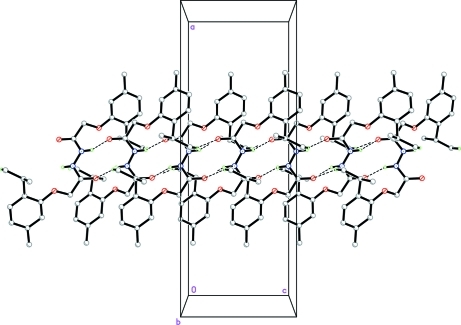
The crystal structure of the title compound, viewed along the b axis. H atoms not involved in hydrogen bonds (dashed lines) have been omitted for clarity.

## supplementary crystallographic information

### Comment

Hydrazides have been demonstrated to possess antimicrobial, anticonvulsant, analgesic, antiinflammatory, antiplatelet, antitubercular, anticancer and antitumoral activities (Bedia *et al.*, 2006; Rollas *et al.*, 2002; Terzioglu & Gürsoy, 2003). These are key intermediates in the preparation of hydrazones. Hydrazones are versatile intermediates and important building blocks. Hydrazones of aliphatic and aromatic methyl ketones yield pyrazole-4-carboxaldehyde on formylation with Vilsmeier reagent (Bratenko *et al.*, 1999). Aryl hydrazones are important building blocks for the synthesis of a variety of heterocyclic compounds such as pyrazolines and pyrazoles (Rai *et al.*, 2008). The condensation of ethyl [5-methyl-2-(propan-2-yl)phenoxy]acetate with hydrazides of corresponding ester in presence of a catalytic amount of sodium acetate yielded the  titled compound. The hydrazides are in turn obtained by refluxing ester with hydrazine hydrate in presence of ethanol .

The title molecule, Fig. 1, is lying across a crystallographic inversion center (symmetry code: -x+1, -y+1, -z). The 1,2-diethylhydrazine moeity (O2/O2A/N1/N1A/C7/C7A/C8/C8A) is nearly planar, with a maximum deviation of 0.024 (1) Å at atoms N1 and N1A, and is inclined at an angle of 54.20 (4)° with the phenyl ring (C1-C6). Bond lengths (Allen *et al.*, 1987) and angles are within normal ranges.

In the crystal packing, the molecules are linked *via* a pair of intermolecular N1–H1N1···O2 hydrogen bonds (Table 1), generating R^2^_2_ (10) ring motifs (Bernstein *et al.*, 1995). The molecules are further linked into one-dimensional chains along [001] *via* adjacent ring motifs and intermolecular C11–H11A···O2 hydrogen bonds (Table 1). The crystal structure is further stabilized by C7—H7B···Cg1 (Table 1) interactions, where Cg1 is the centroid of the C1-C6 phenyl ring.

### Experimental

2-[5-Methyl-2-(propan-2-yl)phenoxy]acethydrazide (0.01 mol) and ethyl [5-methyl-2-(propan-2-yl)phenoxy]acetate (0.01 mol) in ethanol and a catalytic amount of anhydrous sodium acetate was refluxed for 2-3 h. The excess of ethanol was removed by distillation and the reaction mixture was kept overnight. The solid product separated was filtered. It was then recrystallized from ethanol. Colourless needles were obtained from ethanol by slow evaporation.

### Refinement

Atom H1N1 was located from the difference Fourier map and refined freely [N1–H1N1 = 0.902 (15) Å]. The remaining H atoms were positioned geometrically and refined using a riding model with C–H = 0.93-0.97 Å and *U*_iso_(H) = 1.2 or 1.5 *U*_eq_(C). A rotating-group model was applied for the methyl group.

### Crystal data

**Table:**

C_24_H_32_N_2_O_4_	*F*(000) = 888
*M**_r_* = 412.52	*D*_x_ = 1.195 Mg m^−^^3^
Orthorhombic, *P**b**c**n*	Mo *K*α radiation, λ = 0.71073 Å
Hall symbol: -P 2n 2ab	Cell parameters from 9950 reflections
*a* = 23.6018 (8) Å	θ = 3.1–33.9°
*b* = 11.2077 (4) Å	µ = 0.08 mm^−^^1^
*c* = 8.6653 (3) Å	*T* = 100 K
*V* = 2292.16 (14) Å^3^	Needle, colourless
*Z* = 4	0.98 × 0.23 × 0.18 mm

### Data collection

**Table:**

Bruker SMART APEXII CCD diffractometer	3337 independent reflections
Radiation source: fine-focus sealed tube	2946 reflections with *I* > 2σ(*I*)
graphite	*R*_int_ = 0.030
φ and ω scans	θ_max_ = 30.0°, θ_min_ = 1.7°
Absorption correction: multi-scan (*SADABS*; Bruker, 2009)	*h* = −33→33
*T*_min_ = 0.914, *T*_max_ = 0.986	*k* = −15→15
38738 measured reflections	*l* = −12→12

### Refinement

**Table:**

Refinement on *F*^2^	Primary atom site location: structure-invariant direct methods
Least-squares matrix: full	Secondary atom site location: difference Fourier map
*R*[*F*^2^ > 2σ(*F*^2^)] = 0.041	Hydrogen site location: inferred from neighbouring sites
*wR*(*F*^2^) = 0.111	H atoms treated by a mixture of independent and constrained refinement
*S* = 1.04	*w* = 1/[σ^2^(*F*_o_^2^) + (0.0544*P*)^2^ + 0.8669*P*] where *P* = (*F*_o_^2^ + 2*F*_c_^2^)/3
3337 reflections	(Δ/σ)_max_ = 0.001
143 parameters	Δρ_max_ = 0.34 e Å^−^^3^
0 restraints	Δρ_min_ = −0.21 e Å^−^^3^

### Special details

**Table:**

Experimental. The crystal was placed in the cold stream of an Oxford Cryosystems Cobra open-flow nitrogen cryostat (Cosier & Glazer, 1986) operating at 100.0 (1) K.
Geometry. All esds (except the esd in the dihedral angle between two l.s. planes) are estimated using the full covariance matrix. The cell esds are taken into account individually in the estimation of esds in distances, angles and torsion angles; correlations between esds in cell parameters are only used when they are defined by crystal symmetry. An approximate (isotropic) treatment of cell esds is used for estimating esds involving l.s. planes.
Refinement. Refinement of F^2^ against ALL reflections. The weighted R-factor wR and goodness of fit S are based on F^2^, conventional R-factors R are based on F, with F set to zero for negative F^2^. The threshold expression of F^2^ > 2sigma(F^2^) is used only for calculating R-factors(gt) etc. and is not relevant to the choice of reflections for refinement. R-factors based on F^2^ are statistically about twice as large as those based on F, and R- factors based on ALL data will be even larger.

### Fractional atomic coordinates and isotropic or equivalent isotropic displacement parameters (Å^2^)

**Table:**

	*x*	*y*	*z*	*U*_iso_*/*U*_eq_	
O1	0.60698 (3)	0.34104 (6)	0.17205 (7)	0.01862 (15)	
O2	0.56593 (3)	0.42523 (7)	−0.18634 (8)	0.02124 (16)	
N1	0.52505 (3)	0.48416 (8)	0.03709 (9)	0.01797 (17)	
C1	0.62322 (4)	0.26903 (8)	0.42242 (10)	0.01853 (18)	
C2	0.65864 (5)	0.26034 (9)	0.55037 (12)	0.0247 (2)	
H2A	0.6462	0.2184	0.6366	0.030*	
C3	0.71232 (5)	0.31277 (10)	0.55309 (12)	0.0268 (2)	
H3A	0.7350	0.3050	0.6403	0.032*	
C4	0.73205 (4)	0.37621 (9)	0.42683 (12)	0.0239 (2)	
C5	0.69717 (4)	0.38699 (9)	0.29676 (11)	0.02033 (19)	
H5A	0.7098	0.4294	0.2111	0.024*	
C6	0.64369 (4)	0.33435 (8)	0.29522 (10)	0.01671 (18)	
C7	0.62177 (4)	0.41673 (8)	0.04618 (10)	0.01788 (18)	
H7A	0.6498	0.3780	−0.0186	0.021*	
H7B	0.6377	0.4909	0.0843	0.021*	
C8	0.56832 (4)	0.44162 (8)	−0.04601 (10)	0.01595 (17)	
C9	0.56630 (4)	0.20691 (9)	0.41279 (11)	0.02125 (19)	
H9A	0.5411	0.2573	0.3507	0.026*	
C10	0.57351 (5)	0.08855 (11)	0.32753 (16)	0.0363 (3)	
H10A	0.5876	0.1034	0.2254	0.054*	
H10B	0.5376	0.0487	0.3211	0.054*	
H10C	0.5999	0.0391	0.3825	0.054*	
C11	0.53764 (6)	0.18644 (10)	0.56823 (13)	0.0320 (2)	
H11A	0.5353	0.2607	0.6232	0.048*	
H11C	0.5594	0.1303	0.6274	0.048*	
H11B	0.5002	0.1554	0.5520	0.048*	
C12	0.78972 (5)	0.43480 (12)	0.42762 (15)	0.0352 (3)	
H12A	0.8130	0.3984	0.5054	0.053*	
H12B	0.7856	0.5183	0.4496	0.053*	
H12C	0.8072	0.4249	0.3284	0.053*	
H1N1	0.5294 (6)	0.5096 (13)	0.1351 (18)	0.032 (3)*	

### Atomic displacement parameters (Å^2^)

**Table:**

	*U*^11^	*U*^22^	*U*^33^	*U*^12^	*U*^13^	*U*^23^
O1	0.0197 (3)	0.0228 (3)	0.0134 (3)	−0.0028 (2)	−0.0038 (2)	0.0055 (2)
O2	0.0235 (3)	0.0287 (4)	0.0115 (3)	0.0021 (3)	−0.0007 (2)	−0.0008 (3)
N1	0.0171 (3)	0.0263 (4)	0.0106 (3)	0.0022 (3)	−0.0028 (3)	−0.0007 (3)
C1	0.0237 (4)	0.0160 (4)	0.0159 (4)	0.0038 (3)	−0.0018 (3)	0.0012 (3)
C2	0.0354 (5)	0.0215 (4)	0.0173 (4)	0.0048 (4)	−0.0064 (4)	0.0033 (4)
C3	0.0312 (5)	0.0261 (5)	0.0231 (5)	0.0085 (4)	−0.0128 (4)	−0.0026 (4)
C4	0.0205 (4)	0.0257 (5)	0.0256 (5)	0.0057 (4)	−0.0065 (3)	−0.0069 (4)
C5	0.0184 (4)	0.0234 (4)	0.0192 (4)	0.0015 (3)	−0.0014 (3)	−0.0018 (4)
C6	0.0190 (4)	0.0176 (4)	0.0135 (4)	0.0035 (3)	−0.0033 (3)	−0.0006 (3)
C7	0.0180 (4)	0.0227 (4)	0.0130 (4)	−0.0008 (3)	−0.0005 (3)	0.0038 (3)
C8	0.0185 (4)	0.0165 (4)	0.0128 (4)	−0.0018 (3)	−0.0006 (3)	0.0019 (3)
C9	0.0239 (4)	0.0211 (4)	0.0187 (4)	0.0008 (3)	0.0007 (3)	0.0036 (3)
C10	0.0315 (6)	0.0348 (6)	0.0426 (7)	−0.0078 (5)	0.0047 (5)	−0.0164 (5)
C11	0.0442 (6)	0.0270 (5)	0.0247 (5)	−0.0059 (5)	0.0093 (5)	0.0048 (4)
C12	0.0208 (5)	0.0460 (7)	0.0389 (6)	0.0005 (4)	−0.0086 (4)	−0.0107 (5)

### Geometric parameters (Å, °)

**Table:**

O1—C6	1.3767 (10)	C5—H5A	0.9300
O1—C7	1.4252 (11)	C7—C8	1.5189 (12)
O2—C8	1.2311 (11)	C7—H7A	0.9700
N1—C8	1.3375 (11)	C7—H7B	0.9700
N1—N1^i^	1.3922 (14)	C9—C11	1.5246 (14)
N1—H1N1	0.902 (15)	C9—C10	1.5279 (15)
C1—C2	1.3920 (13)	C9—H9A	0.9800
C1—C6	1.4087 (13)	C10—H10A	0.9600
C1—C9	1.5153 (13)	C10—H10B	0.9600
C2—C3	1.3968 (16)	C10—H10C	0.9600
C2—H2A	0.9300	C11—H11A	0.9600
C3—C4	1.3854 (16)	C11—H11C	0.9600
C3—H3A	0.9300	C11—H11B	0.9600
C4—C5	1.4009 (13)	C12—H12A	0.9600
C4—C12	1.5115 (15)	C12—H12B	0.9600
C5—C6	1.3934 (13)	C12—H12C	0.9600
			
C6—O1—C7	118.14 (7)	O2—C8—N1	123.37 (8)
C8—N1—N1^i^	119.41 (9)	O2—C8—C7	122.02 (8)
C8—N1—H1N1	122.2 (9)	N1—C8—C7	114.61 (8)
N1^i^—N1—H1N1	116.8 (9)	C1—C9—C11	114.44 (9)
C2—C1—C6	116.97 (9)	C1—C9—C10	109.08 (8)
C2—C1—C9	122.97 (9)	C11—C9—C10	110.24 (9)
C6—C1—C9	119.98 (8)	C1—C9—H9A	107.6
C1—C2—C3	121.93 (10)	C11—C9—H9A	107.6
C1—C2—H2A	119.0	C10—C9—H9A	107.6
C3—C2—H2A	119.0	C9—C10—H10A	109.5
C4—C3—C2	120.49 (9)	C9—C10—H10B	109.5
C4—C3—H3A	119.8	H10A—C10—H10B	109.5
C2—C3—H3A	119.8	C9—C10—H10C	109.5
C3—C4—C5	118.83 (9)	H10A—C10—H10C	109.5
C3—C4—C12	121.47 (9)	H10B—C10—H10C	109.5
C5—C4—C12	119.69 (10)	C9—C11—H11A	109.5
C6—C5—C4	120.22 (9)	C9—C11—H11C	109.5
C6—C5—H5A	119.9	H11A—C11—H11C	109.5
C4—C5—H5A	119.9	C9—C11—H11B	109.5
O1—C6—C5	123.67 (8)	H11A—C11—H11B	109.5
O1—C6—C1	114.77 (8)	H11C—C11—H11B	109.5
C5—C6—C1	121.55 (8)	C4—C12—H12A	109.5
O1—C7—C8	107.97 (7)	C4—C12—H12B	109.5
O1—C7—H7A	110.1	H12A—C12—H12B	109.5
C8—C7—H7A	110.1	C4—C12—H12C	109.5
O1—C7—H7B	110.1	H12A—C12—H12C	109.5
C8—C7—H7B	110.1	H12B—C12—H12C	109.5
H7A—C7—H7B	108.4		
			
C6—C1—C2—C3	−0.57 (14)	C9—C1—C6—O1	3.50 (12)
C9—C1—C2—C3	176.25 (9)	C2—C1—C6—C5	0.57 (13)
C1—C2—C3—C4	0.24 (16)	C9—C1—C6—C5	−176.35 (8)
C2—C3—C4—C5	0.13 (15)	C6—O1—C7—C8	−161.38 (7)
C2—C3—C4—C12	179.36 (10)	N1^i^—N1—C8—O2	−2.20 (16)
C3—C4—C5—C6	−0.14 (14)	N1^i^—N1—C8—C7	176.73 (10)
C12—C4—C5—C6	−179.38 (9)	O1—C7—C8—O2	−127.62 (9)
C7—O1—C6—C5	−7.22 (13)	O1—C7—C8—N1	53.43 (10)
C7—O1—C6—C1	172.93 (8)	C2—C1—C9—C11	28.23 (13)
C4—C5—C6—O1	179.93 (9)	C6—C1—C9—C11	−155.04 (9)
C4—C5—C6—C1	−0.23 (14)	C2—C1—C9—C10	−95.77 (12)
C2—C1—C6—O1	−179.58 (8)	C6—C1—C9—C10	80.96 (11)

Symmetry codes: (i) −*x*+1, −*y*+1, −*z*.

### Hydrogen-bond geometry (Å, °)

**Table:**

Cg1 is the centroid of the C1–C6 phenyl ring.

**Table:**

*D*—H···*A*	*D*—H	H···*A*	*D*···*A*	*D*—H···*A*
N1—H1N1···O2^ii^	0.902 (16)	1.916 (15)	2.7759 (11)	158.8 (13)
C11—H11A···O2^iii^	0.96	2.58	3.4830 (14)	157
C7—H7B···Cg1^iv^	0.97	2.68	3.3706 (10)	129

Symmetry codes: (ii) *x*, −*y*+1, *z*+1/2; (iii) *x*, *y*, *z*+1; (iv) −*x*−1/2, *y*+1/2, *z*−1.
